# Identification of genes controlling grain size in barley through RNA-seq and weighted gene co-expression network analysis

**DOI:** 10.1007/s10142-026-01908-x

**Published:** 2026-06-11

**Authors:** Rundong Xie, Yan Wang, Mingqi Yang, Jiahao Zhou, Youhua Yao, Yongmei Cui, Xin Li, Baojun Ding, Xiaohua Yao, Kunlun Wu

**Affiliations:** 1 Qinghai Academy of Agricultural and Forestry Sciences, Qinghai Univeisity, Xining, Qinghai China; 2 College of Agriculture and Animal Husbandry, Qinghai University, Xining, Qinghai China; 3 Qinghai Key Laboratory of Hulless Barley Improvement/Qinghai Subcenter of National Hulless Barley Improvement/Laboratory for Research and Utilization of Qinghai Tibet Plateau Germplasm Resources, Xining, Qinghai China

**Keywords:** Barley, Grain size, Rna-seq, WGCNA, Candidate genes

## Abstract

To elucidate the molecular mechanisms underlying grain size formation in barley, this study selected two distinct barley cultivars with significant differences in grain dimensions: the large-grained cultivar ‘jianglinhuang’ and the small-grained cultivar ‘heiseyeqingke’. We conducted a comprehensive comparative analysis by integrating phenotypic measurements with RNA-seq data to elucidate molecular-level differences between barley varieties with distinct grain sizes. The study focused on three critical grain developmental stages. Dynamic phenotypic monitoring revealed that grain length and thickness exhibited significant differences across all three stages, whereas grain width only showed significant variation during the mid- and late-filling stages. Transcriptome analysis revealed that the number of Differentially Expressed Genes(DEGs) peaked during the mid-filling stage. Subsequent Weighted Gene Co-expression Network Analysis (WGCNA) revealed a brown module highly associated with grain size traits. By intersecting the module’s hub genes with pan-stage DEGs, we obtained 13 candidate genes. Following homology-based screening and functional annotation, five of these candidate genes were found to be orthologs of known regulators of grain size in rice and other crops, and were therefore designated as the core candidate genes for this study. The RT-qPCR validation results further confirmed the stage and cultivar-specific expression patterns of these genes, supporting their potential as molecular targets for barley grain improvement. Through an integrated multi-omics approach, this study has identified a set of key genes potentially governing grain size and yield potential in barley, providing novel insights into the molecular mechanisms of grain formation and laying the groundwork for future genetic improvement.

**Supplementary Information:**

The online version contains supplementary material available at 10.1007/s10142-026-01908-x.

## Introduction

Barley (*Hordeum vulgare* L.), one of the earliest domesticated cereals, is globally cultivated due to its remarkable adaptability and diverse applications(Pankin et al. 2018). Barley is one of the most important cereal crops in terms of economic importance, following rice, wheat, and corn(Newton et al. 2011). On the Qinghai-Tibet Plateau, barley serves as a primary staple crop for the local population(Wang et al. 2023). Furthermore, barley holds significant value in malting and brewing(Kihara et al. 2024), food processing(Al-Asmari et al. 2025), and animal feed production (Ibrahim et al. 2023). Consequently, developing high-yielding, stable-performance barley varieties for high-altitude regions is crucial for ensuring food security and increasing the income of local farmers and herders. Faced with a progressively widening gap between market supply and demand, it is imperative to intensify efforts to enhance both the yield and quality of barly.

Grain size is a complex quantitative trait governed by multiple genes and factors, which has remained a major focus of research and breeding efforts since the advent of modern agriculture(Gasparis and Miloszewski 2023). The rapid advancement of high-throughput sequencing technologies in recent years has facilitated the identification of numerous genes and loci associated with grain size across multiple crop species. In the staple food crop rice, genes such as *OsSPL4*(Hu et al. 2021), *OsRac1*(Zhang et al. 2019), and *OsARaf25*(Zhang et al. 2025) have been functionally characterized as key regulators of grain dimensions. Research in *Arabidopsis* has also identified key seed size regulators, including *AtTTG1*(Luo et al. 2025), *AtENO2*(Liu et al. 2020). To date, researchers have identified several conserved signaling pathways governing seed size regulation in model plants such as *Arabidopsis* and rice, including the IKU (HAIKU) pathway, ubiquitin-proteasome pathway, G-protein (GTP-binding protein) signaling, mitogen-activated protein kinase (MAPK) cascades, phytohormone signaling networks, and transcriptional regulatory mechanisms(Hong et al. 2023). The elucidation of these regulatory mechanisms offers significant mechanistic insights into grain development, paving the way for molecular breeding strategies aimed at crop enhancement.

Barley grain size is a key agronomic trait that directly affects yield and quality. However, the research on its genetic regulation mechanism is still not perfect. Studies have shown that quantitative trait loci (QTLs) regulating grain size were mapped on all seven chromosomes of barley(Hong et al. 2023; Wang et al. 2019, 2021). To precisely locate these sites, studies have utilized omics technologies such as genome-wide association studies (GWAS) and RNA-seq to successfully identify a large number of trait-associated sites and candidate genes. These candidate genes are involved in various pathways including transcriptional regulation, hormone signaling, and metabolism, achieving a breakthrough from macro-level sites to specific candidate genes(Hong et al. 2024; Luan et al. 2025). A study precisely located the locus *qGL2H* affecting grain length by constructing a DH population(Watt et al. 2020). Additionally, through reverse genetics approaches utilizing technologies such as CRISPR, studies have confirmed the association of several genes with grain size. For instance, *HvDep1*, *HvGL7-2 H*, *GW2* and *D-hordein* have all been demonstrated to be involved in the determination of barley grain size(Kis et al. 2024; Liu et al. 2025; Wendt et al. 2016; Yang et al. 2020). Furthermore, studies employing comparative transcriptomics and network analysis, using methods such as WGCNA, have systematically screened numerous candidate genes potentially associated with barley grain size in differential varieties(Kong et al. 2022; Wang et al. 2025). A recent study demonstrated that overexpression of *HvPR1* in barley can significantly enhance thousand kernel weight (TKW) and grain hardness, thereby providing a novel target for yield improvement(Wang et al. 2026). Despite the aforementioned achievements, current research still faces significant limitations. On the one hand, a large number of reported candidate genes still lack functional validation, and their upstream and downstream regulatory mechanisms remain unclear. On the other hand, most existing studies focus on static gene screening or the functional characterization of individual genes, failing to systematically elucidate the transcriptional regulatory basis underlying trait differences among materials with diverse genetic backgrounds from the holistic perspectives of dynamic temporal processes during grain development and gene interaction networks.

To address the aforementioned challenges, this study aims to employ a dynamic comparative transcriptomics strategy to systematically unravel the temporal regulatory network basis underlying grain size variation. This study utilized two barley cultivars exhibiting significant differences in grain traits as experimental materials. Based on key developmental stages of dimensional establishment, the grain filling process was categorized into three distinct phases: the early stage predominated by cell proliferation, the mid stage characterized by dry matter accumulation and grain expansion, and the late stage entering maturation and stabilization(Wang et al. 2025). Grain samples from these three developmental stages were respectively collected for RNA-seq analysis, with simultaneous measurement of phenotypic parameters including grain length (GL), grain width (GW), and grain thickness (GT). An integrated analysis was then performed by combining the phenotypic data from all three stages with the corresponding gene expression profiles. We subsequently leveraged the multi-stage phenotypic datasets and transcriptomic profiles to conduct WGCNA, which employed co-expression network topology to screen for candidate genes implicated in grain size determination. This study employed a comparative transcriptomics strategy to systematically elucidate the differences and commonalities in the regulatory networks and the expression of key genes governing grain size among different barley cultivars. Our findings not only advance the understanding by elucidating the genetic diversity underlying barley grain development, but also provide specific molecular targets and a solid theoretical foundation for the targeted breeding of high-yielding and superior-quality barley cultivars.

## Materials and methods

### Sample collection

This study employed two six-rowed barley cultivars with distinct grain phenotypes as experimental materials: the large-grained cultivar ‘jianglinhuang’ and the small-grained cultivar ‘heiseyeqingke’. Both cultivars were obtained from the National Germplasm Bank of the Chinese Academy of Agricultural Sciences and cultivated in the experimental field of the College of Agriculture and Forestry, Qinghai University (36.73°N, 101.75°E) in April 2024. Grain samples were collected at three key developmental stages. According to the Zadoks growth scale for cereal development(Zadoks 1985), these stages were defined as the early milk stage, late milk stage, and soft dough stage, at 2, 3, and 5 weeks after anthesis, respectively. These three stages represent the principal phases during which final grain size is determined in barley. For each sampling time point, a minimum of three biological replicates were established. Immediately following collection, all samples were preserved in liquid nitrogen and subsequently transported to Shanghai Majorbio Bio-pharm Technology Co, Ltd for subsequent analysis. The remaining samples were stored at −80 °C for subsequent RT-qPCR analysis.

### Data collection

GL, width, and GT data were collected and analyzed from a random sample of at least 500 intact grains from each developmental stage of both cultivars. Measurements were performed using the Wanshen SC-G Automated Grain Testing Analyzer (Hangzhou Wanshen Detection Technology Co., Ltd., Hangzhou, China).

### RNA extraction and transcriptome sequencing

Total RNA was extracted from grains at three grain-filling stages using TRIzol reagent according to the manufacturer’s instructions. RNA purity and concentration were determined using a NanoDrop 2000 spectrophotometer (Thermo Fisher Scientific, Waltham, MA, USA), while RNA integrity was assessed with an Agilent 5300 Bioanalyzer (Agilent Technologies, Santa Clara, CA, USA). Sequencing libraries were constructed with the VAHTS Universal V5 RNA-seq Library Prep Kit following the manufacturer’s protocol and sequenced on the Illumina NovaSeq 6000 platform to generate 150-bp paired-end reads. Raw reads in FASTQ format were processed with FASTP(Chen et al. 2018)to remove low-quality sequences, yielding clean reads for subsequent analyses. Both transcriptome sequencing and bioinformatic analyses were performed by Majorbio Bio-pharm Technology Co., Ltd. (Shanghai, China).

The reference genome utilized in this study was *Hordeum vulgare* (barley), assembly version MorexV3. The genomic sequences and annotations were sourced from the Ensembl Plants database.

MorexV3(http://plants.ensembl.org/Hordeum_vulgare/Info/Index).

### Gene expression analysis and differential gene screening

In RNA-Seq analysis, gene expression levels are quantified based on transcript abundance, whereby a higher number of clean reads mapped to a specific genomic region indicates a higher expression level of the corresponding gene. In this study, clean reads were aligned to the reference genome using HiSat2, followed by quantification of gene and transcript expression levels with RSEM software. These quantification results provided the foundation for subsequent differential expression analysis. Based on the gene read counts obtained from RSEM quantification, we performed differential expression analysis across multiple samples using DESeq2 software to identify genes exhibiting significant expression changes under different conditions. The screening criteria for differentially expressed genes were established as follows: false discovery rate (FDR) < 0.05 and |log_2_FC| ≥ 1 (corresponding to a fold change of FC ≥ 2 or FC ≤ 0.5). Here, Fold Change (FC) represents the expression ratio between two sample groups, while log_2_FC denotes its log_2_-transformed value, with a larger absolute value indicating a more significant difference. The FDR is derived from correcting the p-value for statistical significance and serves to control the false positive rate. By integrating the results of expression quantification and differential analysis, combined with functional annotation information, a foundation is established for further elucidating the underlying gene regulatory mechanisms.

DESeq2(http://bioconductor.org/packages/stats/bioc/DESeq2/).

HiSat2(http://ccb.jhu.edu/software/hisat2/index.shtml).

RSEM(http://deweylab.github.io/RSEM/).

### Principal component analysis

Principal Component Analysis (PCA) was performed to evaluate the overall transcriptional differences among samples and the reproducibility of biological replicates. The analysis included all samples encompassing biological replicates from three grain developmental stages of both barley cultivars. PCA was conducted based on read counts of all expressed genes to visualize natural clustering patterns among samples.

### Differential gene GO(gene ontology) and KEEG༈kyoto encyclopedia of genes and genomes༉ enrichment analysis

GO enrichment analysis was performed using the Goatools software (https://github.com/tanghaibao/GOatools) with Fisher’s exact test. To control the false positive rate, four multiple test correction methods (Bonferroni, Holm, Sidak, and false discovery rate) were applied for p-value adjustment. A GO term was considered significantly enriched when the FDR-adjusted p-value (p_fdr) was < 0.05.

KEGG pathway enrichment analysis of differentially expressed genes was performed using the Python SciPy package (https://scipy.org/install/). The computational principle was consistent with the GO functional enrichment analysis, employing Fisher’s exact test. To control the false positive rate, the Benjamini-Hochberg (FDR) method was applied for multiple testing correction. KEGG pathways with corrected p-values < 0.05 were considered significantly enriched. To control the false discovery rate, the Benjamini-Hochberg (FDR) method was applied for multiple testing correction. KEGG pathways with corrected p-values (FDR) < 0.05 were defined as significantly enriched pathways in the differentially expressed gene set.

### Weighted gene co-expression network analysis (WGCNA)

Gene selection was performed using RSEM software, applying the criteria of mean expression > 1 and coefficient of variation > 0.1 as filtering thresholds. To construct a biologically meaningful co-expression network approximating scale-free topology, a soft threshold power of 9 was selected, achieving approximately 85% model fit. Gene modules were identified using dynamic tree cutting algorithm with a minimum module size of 30 genes. A cut height of 0.25 was set, and co-expression modules with similar clustering patterns were merged, yielding candidate modules most significantly associated with GL, GW, and GT.

### Identification of hub and candidate genes

Hub genes within each module were identified through visual network analysis. The top 30 nodes ranked by intramodular connectivity were selected for further investigation. The top 30 hub genes were intersected with the DEGs to obtain the overlapping genes. The functional annotation of these overlapping genes was then performed to identify the final candidate genes for this study.

### Validation of candidate genes by RT-qPCR

qRT-PCR experiments followed MIQE guidelines(Bustin 2024). Quantitative primers were designed with Primer 5.0 (Supplemental Table S1). Grains from both Heiseyeqingke and Jianglinhuang barly, collected at the early, mid, and late filling stages, served as the source for cDNA templates. 18SrRNA was used as the internal reference gene for normalization. cDNA was synthesized using the TaKaRa PrimeScript RT reagent kit (Takara Biomedical Technology Co., Ltd., Beijing, China) in strict accordance with the manufacturer’s instructions. All incubation times, temperatures, and procedural steps were strictly adhered to according to the manufacturer’s specifications. Quantitative real-time reverse transcription polymerase chain reaction (qRT-PCR) was performed using the 2× ChamQ SYBR qPCR Master Mix (Takara Biomedical Technology Co., Ltd., Beijing, China) following the manufacturer’s protocol. The qPCR reaction system consisted of 7.5 µL ddH₂O, 1 µL each of forward and reverse primers, 3 µL cDNA template, and 6.25 µL 2× ChamQ SYBR qPCR Master Mix, comprising a total reaction volume of 20 µL. The qPCR protocol was as follows: initial denaturation at 95 °C for 30 s, followed by 40 cycles of denaturation at 98 °C for 5 s and annealing/extension at 56 °C for 30 s, with a final extension at 95 °C for 10 s. Three biological replicates were included in the experiment and each biological replicate was analyzed in technical triplicate. The qPCR amplification and detection were carried out on a LightCycler 480 System (Roche, Switzerland).

### Yeast two-hybrid (Y2H) verification of *HvSRP_Z7*

For the yeast two-hybrid (Y2H) assay, pGBKT7-SRP_Z7 was used as the bait protein construct, while *HvHor*(HORVU.MOREX.r3.1HG0001110.1) was constructed as pGADT7-HvHor to serve as the prey protein. Yeast co-transformation was performed following the PEG/LiAc method.After co-transformation, the resuspension was spread onto SD/-Leu/-Trp (DDO) dropout medium and incubated at 30 °C for 2–3 days to screen for yeast clones successfully co-transformed. Single colonies were then picked for expanded culture, and the autoactivation activity of the bait protein was assessed by spot assay on SD/-Trp medium.To verify the specific protein–protein interaction, strains confirmed to be free of autoactivation were spotted onto stringent SD/-Trp/-His/-Ade (QDO) medium and incubated at 30 °C for 2–3 days. Combinations that exhibited positive growth exclusively on QDO medium and tested negative for autoactivation were confirmed to exhibit a specific protein-protein interaction.

### Bimolecular fluorescence complementation (BiFC) analysis of the HvSRP_Z7 Protein

The target fragments of P2YN and P2YC were digested with the restriction endonucleases Spe I and Pac I. The target gene inserts were ligated into the digested P2YN and P2YC vectors using T4 DNA ligase, yielding the HvSRP_Z7-YN and HvHor-YC constructs. For the BiFC assay, a previously confirmed interacting protein pair (HvnF3’H-P2YN and HvnKMT-P2YC) was used as a positive control to validate the functionality of the BiFC system(Chen et al. 2024). The empty P2YN and empty P2YC vectors were co-expressed as a negative control to exclude non-specific background fluorescence. The constructs were then transformed into DH5α competent cells (Sangon Biotech, Shanghai, China). Three single positive colonies were selected and sent for sequencing at TsingKe Biotechnology Co., Ltd. (TsingKe, Xi’an, China). Following plasmid extraction from the positive clones, the recombinant plasmids HvSRP_Z7-YN and Hor-YC were introduced into *Agrobacterium tumefaciens* strain GV3101 (Sangon Biotech, Shanghai, China) for Agrobacterium-mediated tobacco transformation experiments. Yellow fluorescent protein (YFP) expression was detected using a laser scanning confocal microscope (Nikon-A1R, Nikon, Shanghai, China).

## Results

### Transcriptome data analysis

#### Dynamic changes in grains during the filling stage

**Fig. 1:**
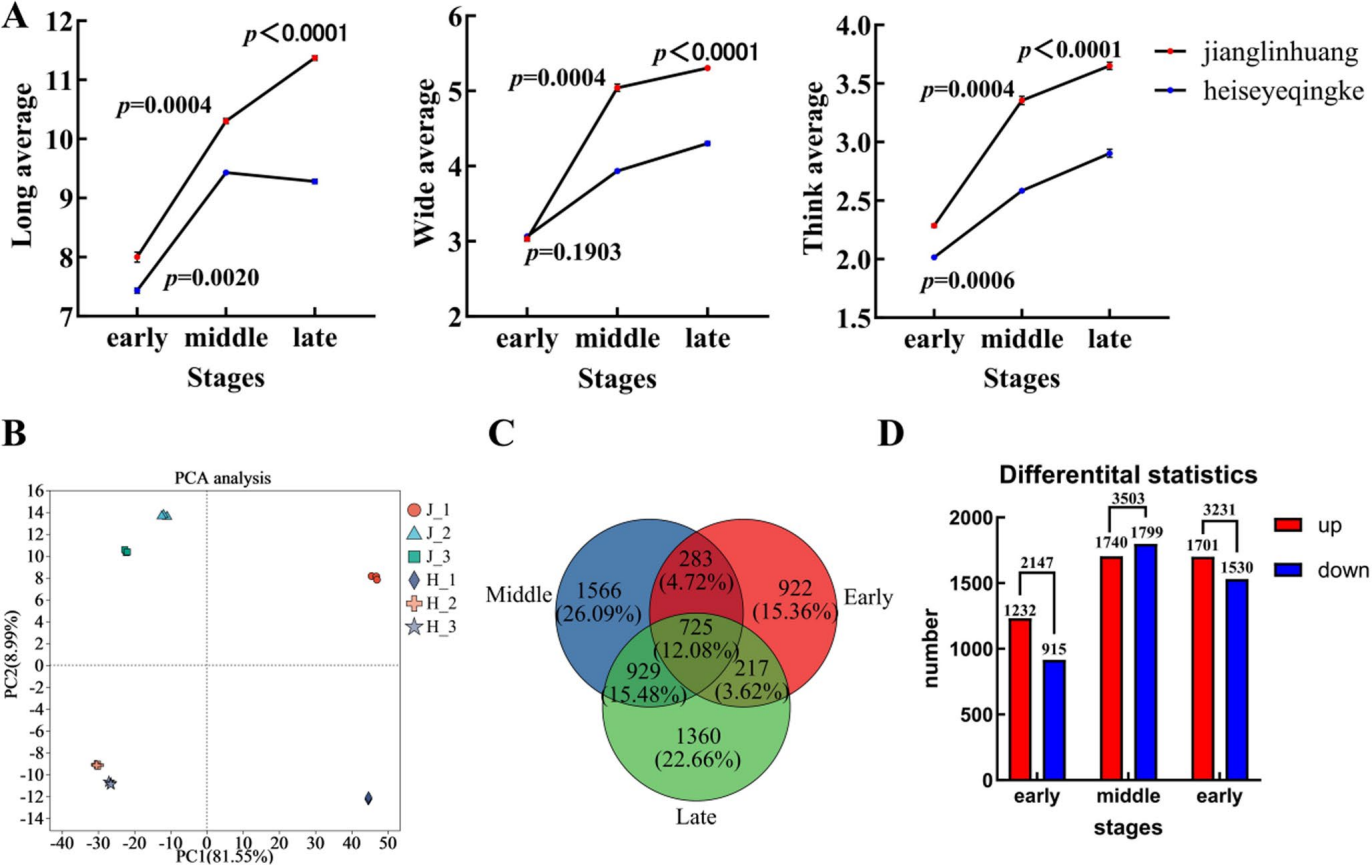
Phenotypic and transcriptomic analysis of barley grains during filling stages. **A**, Grain morphological measurements of two barley cultivars at different developmental stages: measurement of grain length (left), grain width (middle), and grain thickness (right). **B**, PCA of all samples. **C**, Venn diagram displaying the distribution of DEGs. **D**, Histogram showing the number of DEGs

We first performed morphological measurements of grains from the investigated cultivars at different filling stages (Fig. 1A). The results demonstrated rapid grain expansion following the initial filling phase, characterized by substantial increases in GL, GW, and GT. For the large-grained cultivar, GL, GW, and GT maintained a consistent increasing trend throughout the pre-, mid-, and late-filling stages, peaking at the final phase of grain development. In the small-grained cultivar, GW and GT followed a growth pattern comparable to that of the large-grained type. In contrast, GL exhibited a distinct pattern, increasing from the early to mid-filling stage but decreasing thereafter into the late filling stage. At the completion of the grain-filling period, significant differences were observed between the two cultivars in all three dimensions: grain length, width, and thickness.

#### RNA-seq data quality assessment

To investigate the grain development mechanisms in two distinct barley cultivars (‘jianglinhuang’ and ‘heiseyeqingke’), transcriptome sequencing was performed across three key developmental stages of barley grains. Transcriptome sequencing generated 86.16 Gb of clean data from 18 samples in this reference-based transcriptome study. Each sample yielded over 4.50 Gb of clean data, with Q30 scores exceeding 95.65% (Supplemental Table S2) and average GC content ranging from 49.73% to 51.16%. These results demonstrate that the sequencing data are of high quality, ensuring their suitability for subsequent transcriptome analyses.

#### Alignment to the reference genome

Alignment of clean reads to the reference genome yielded mapping rates of 92.31–95.91% (Supplemental Table S3), confirming data reliability and appropriateness for subsequent transcriptomic analyses.

#### PCA of sample expression profiles

We conducted transcriptome sequencing of grain samples across multiple filling stages. Following quality control, all samples demonstrated high-quality metrics with Q30 scores above 95.65%, confirming their reliability for downstream analyses. PCA was conducted to compare grain samples from both cultivars across developmental stages, assessing inter-sample expression heterogeneity and detecting potential outliers (Fig. 1-B). The results showed a clear spatial separation between the two cultivars during the early filling stage, indicating substantial divergence in their transcriptional profiles. In contrast, the two barley cultivars exhibited closer clustering during the mid- and late-filling stages, indicating more convergent transcriptional patterns in these later developmental phases.

#### Differential expression analysis

In this study, a total of 35,826 genes were detected (Supplemental Table S4). To investigate the transcriptional changes between large- and small-grained barley cultivars, we identified and analyzed DEGs across different grain-filling stages in both genotypes. Across the three grain-filling stages, we identified 1,232 up-regulated and 915 down-regulated genes in the early stage, 1,704 up-regulated and 1,799 down-regulated genes in the mid stage, and 1,701 up-regulated and 1,530 down-regulated genes in the late stage, yielding a total of 6,002 differentially expressed genes (Figs. 1C, D). The number of DEGs reached its peak during the mid-filling stage, suggesting this period is particularly critical for transcriptional regulation associated with grain development. The number of DEGs reached its peak during the mid-filling stage, followed by a modest decline in the late filling stage. This temporal pattern suggests that the genetic regulators of grain size are predominantly active during the mid to late filling stages of development.

### Differential gene GO and KEEG enrichment analysis

**Fig. 2:**
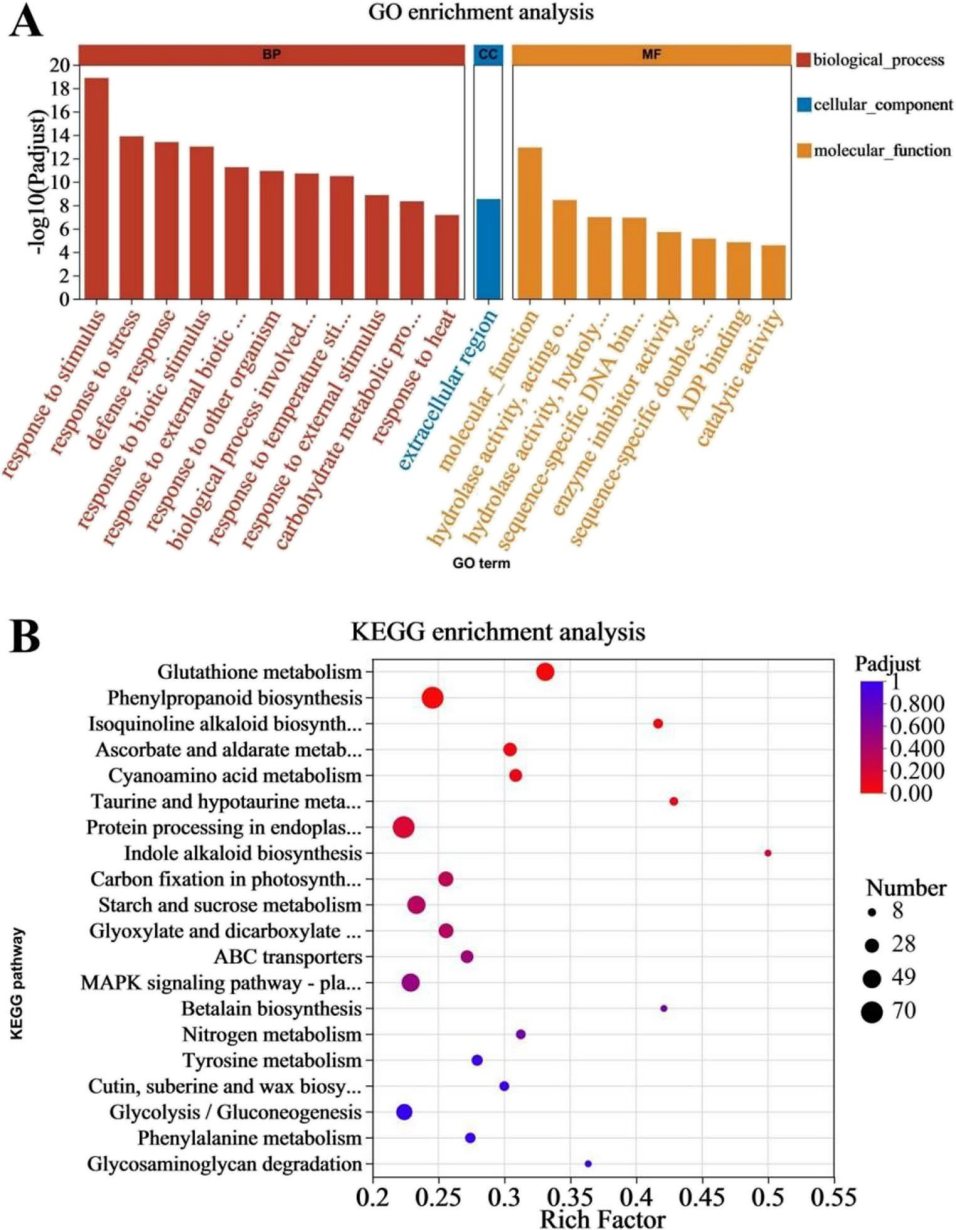
Functional enrichment analysis of differentially expressed genes. **A**, GO enrichment analysis. **B**, KEGG pathway enrichment analysis

To elucidate the biological functions of these 6,003 DEGs, GO enrichment analysis was performed (Supplemental Table S5). The GO enrichment analysis yielded 482 significantly enriched terms, comprising 271 in biological process (BP), 30 in cellular component (CC), and 180 in molecular function (MF). Within the BP category, “response to stimulus” was the most prominent term, encompassing 741 genes. For CC, “extracellular region” achieved the highest enrichment score, containing 208 genes. In the MF category, the term “molecular function” demonstrated the most significant enrichment (Fig. 2-A). To further elucidate the biological functions and interactions of the identified genes, we performed KEGG pathway enrichment analysis (Fig. 2-B, Supplemental Table S6). The three most significantly enriched pathways were “Glutathione metabolism,” “Phenylpropanoid biosynthesis,” and “Isoquinoline alkaloid biosynthesis,” all showing statistically significant associations with the target traits. Notably, the phenylpropanoid biosynthesis pathway contained the highest number of associated genes (70), followed by glutathione metabolism (49 genes) and isoquinoline alkaloid biosynthesis (15 genes).

### WGCNA and co-expression visualization

**Fig. 3:**
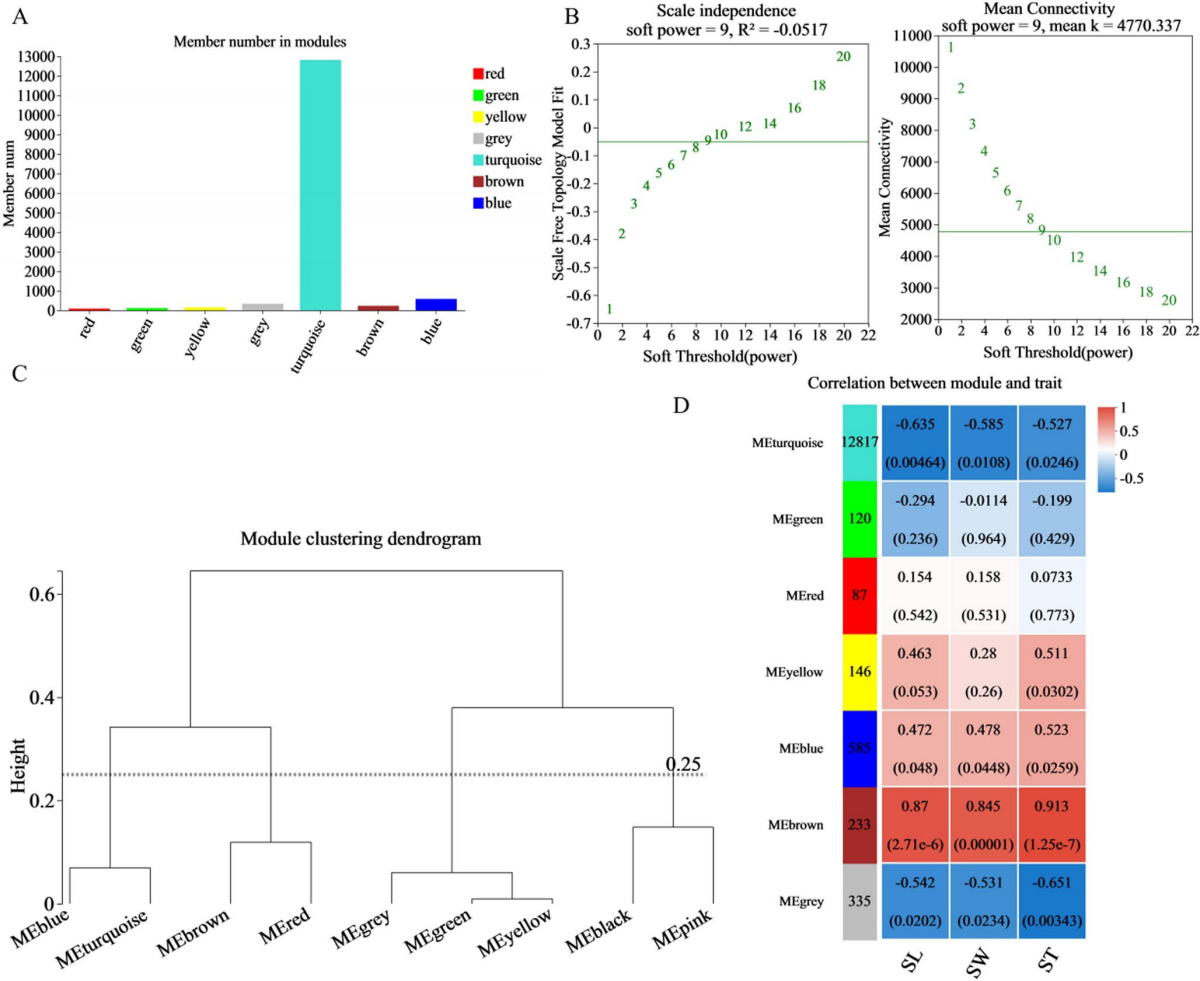
WGCNA. **A**, Module membership statistics. **B**, Left: Scale-free topology fit analysis. The x-axis represents the soft-thresholding power (*β*); the y-axis shows the corresponding scale-free topology model fit (R²). A higher R² value indicates a network closer to scale-free topology. Right: Mean connectivity analysis. The x-axis indicates the soft-thresholding power (*β*); the y-axis displays the mean connectivity (k) of nodes in the corresponding adjacency matrix-transformed network. **C**, Module clustering dendrogram. Each branch represents a module, with closer branch proximity indicating higher similarity between modules, serving as the basis for module merging. The y-axis represents clustering distance. **D**, Module-trait relationship heatmap. The heatmap illustrates correlations between modules and specific traits. The x-axis represents different traits; the y-axis shows different modules. Numbers in the leftmost column indicate gene counts per module. Data on the right side of each cell display correlation coefficients between modules and traits, with significance *p*-values in parentheses. Larger absolute values indicate stronger correlations. Orange indicates negative correlations; purple indicates positive correlations

WGCNA was performed to investigate the correlations between sample expression profiles and phenotypic traits. Following the removal of low-expression genes (FPKM < 1), a total of 14,233 genes were retained for co-expression network construction (Fig. 3-A). To satisfy the scale-free network criterion, it was necessary to determine an appropriate soft-thresholding power (*β*) for the adjacency matrix. For soft-thresholding powers (*β*) ranging from 1 to 30, the scale-free topology fit indices and mean connectivity values were calculated to evaluate network prope Based on the simulation results for *β* values ranging from 1 to 30, the gene expression correlation coefficient and mean connectivity curve approached 1 (indicating an inflection point) at *β* = 9. Therefore, a soft-thresholding power of *β* = 9 was selected to construct the gene co-expression network (Fig. 3-B).

Following the conversion of filtered FPKM values into a topological overlap matrix (TOM), hierarchical clustering was performed for all genes. Co-expression modules were subsequently identified through dynamic tree cutting. A minimum module size of 30 genes was set for the clustering analysis. Modules with a cut height below 0.25 were merged (Fig. 3C). Following this, module-trait relationships were analyzed by calculating correlations between module eigengenes and the trait matrix comprising GL, GW, and GT. A total of seven co-expression modules were identified. Among these, the brown module demonstrated the highest and most significant correlations with all three grain dimensions: grain length (*r* = 0.87, *p* < 0.05), grain width (*r* = 0.845, *p* < 0.05), and grain thickness (*r* = 0.913, *p* < 0.05) (Fig. 3D). The brown module showed the highest correlation with GL, GW, GT, and contained 233 genes (Supplemental Table S7).

### Module visualization and candidate gene screening

**Fig. 4:**
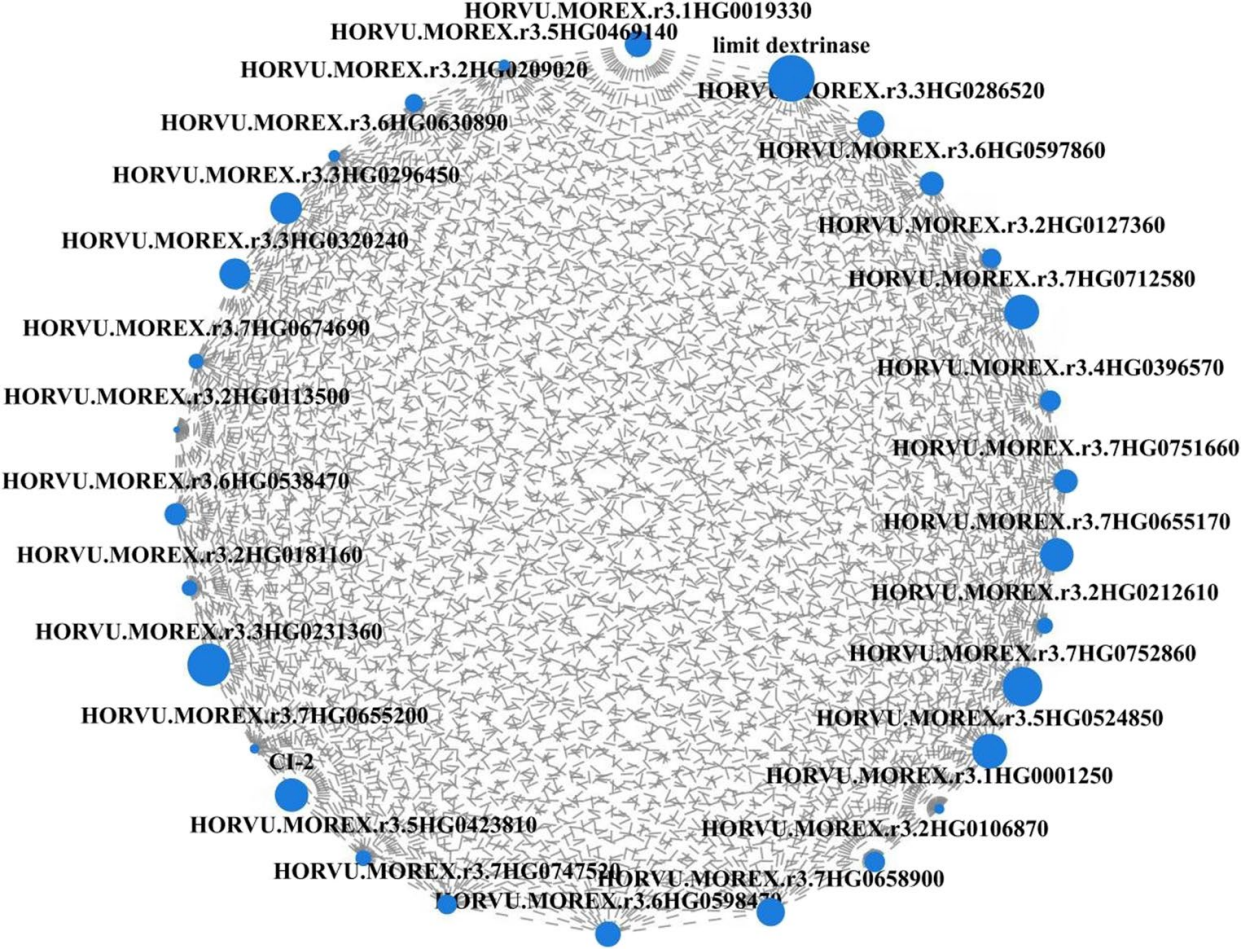
Visualization of the top 30 hub gene co-expression network

Through analysis of genes within the brown module, the top 30 hub genes were identified based on intramodular connectivity values (Fig. 4). The intersection of the 30 hub genes with the DEGs yielded a set of 13 overlapping genes (Supplemental Table S8). Based on functional annotation of the 13 candidate genes, we identified five high-priority candidates: *HvSCPL* (Serine carboxypeptidase, HORVU.MOREX.r3.1HG0019330), *HvTSJT1* (Stem-specific protein, HORVU.MOREX.r3.2HG0209020), *HvSufS* (Cysteine desulfurase, HORVU.MOREX.r3.3HG0286520), *HvEXPA* (Expansin, HORVU.MOREX.r3.4HG0396570), and *HvSRP_Z7* (Serpin, HORVU.MOREX.r3.5HG0524850) (Supplementary Table 3).

### Validation of candidate genes by qRT-PCR

**Fig. 5:**
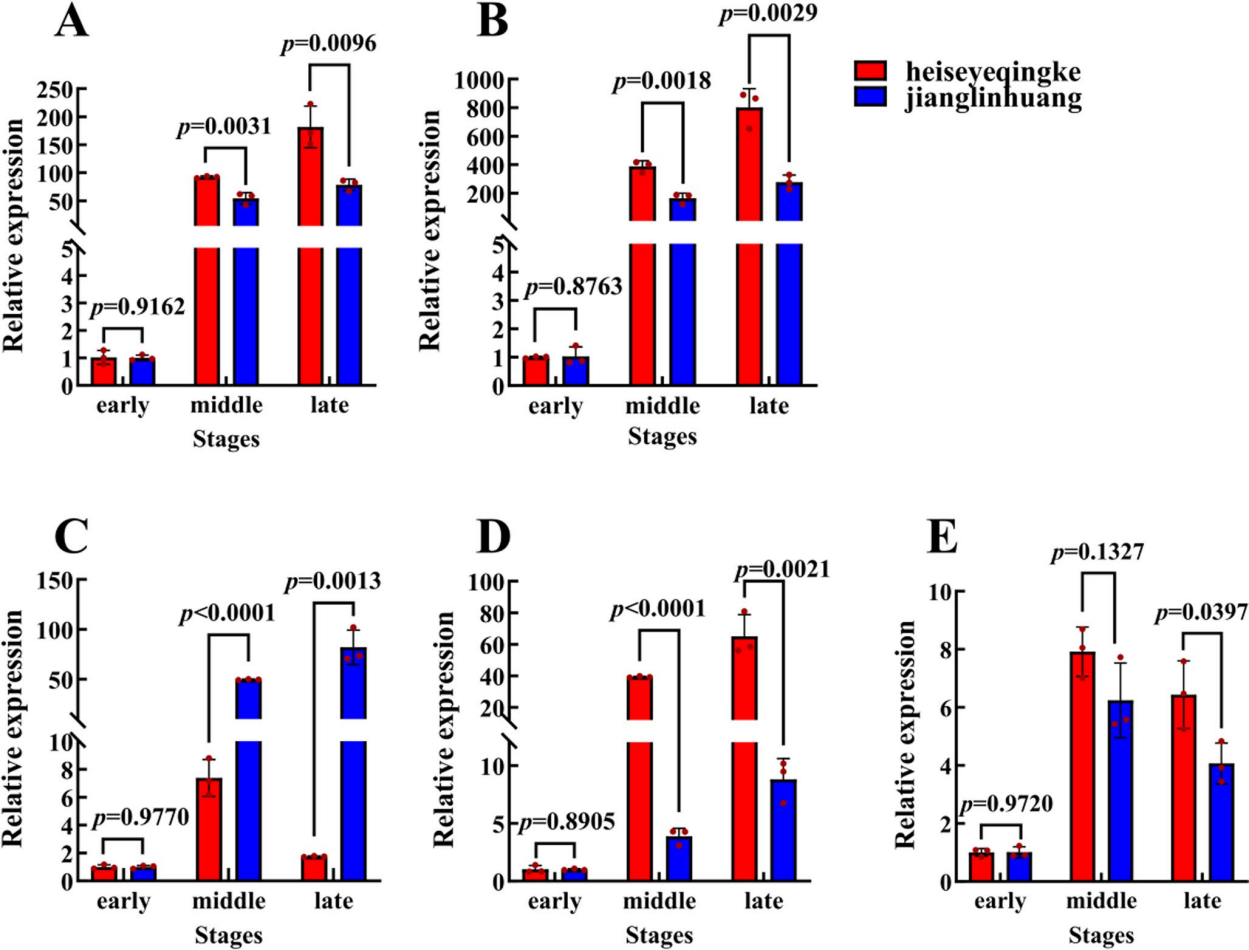
RT-qPCR validation of five candidate genes. **A**, *HvSRP_Z7*, **B**, *HvEXPA*, **C**, *HvSufS*, **D**, *HvTSJT1*, E, *HvSCPL*

To further validate the reliability of the RNA-seq data and the expression patterns of the 13 candidate genes, five genes were selected for qRT-PCR analysis. The experimental results showed a high consistency with the RNA-seq data (Fig. 5,Supplemental Table S9).

### Interaction between *HvSRP_Z7* and *HvHor*

**Fig. 6:**
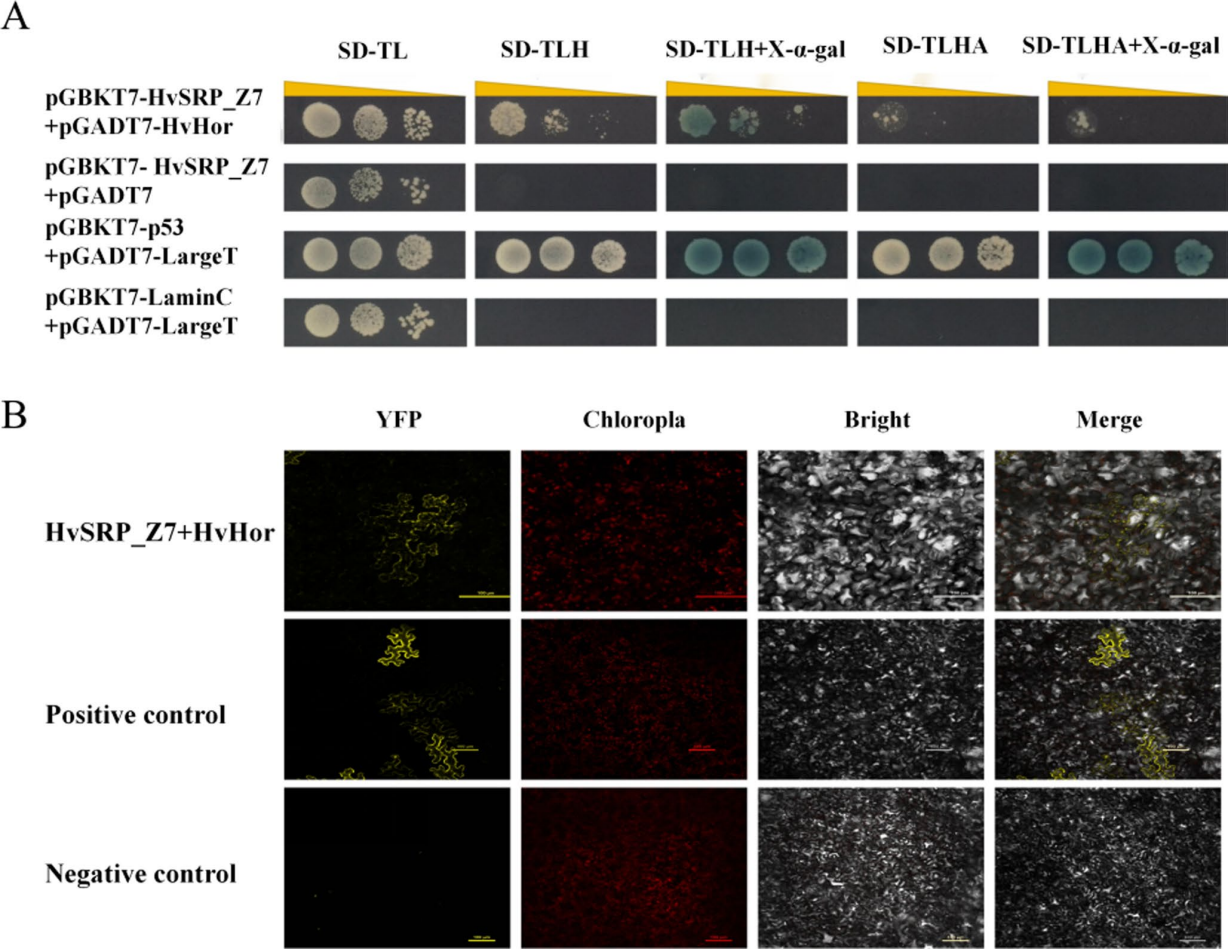
Y2H and BiFC Analysis. **A**, Interaction between *HvSRP_Z7* and *HvHor*, Bacterial suspensions at concentrations of 10⁻¹, 10⁻², 10⁻³, and 10⁻⁴ were spotted onto DDO/X and TDO/X media. DDO/X: SD/-Leu/-Trp/X-α-gal; TDO/X: SD/-Leu/-Trp/-His/X-α-gal. **B**, Interaction between *HvSRP_Z7* and *HvHor* detected by BiFC assay. HvnF3’H-P2YN and HvnKMT-P2YC was used as a positive control to validate the functionality of the BiFC system. Co-expression of the empty P2YN and empty P2YC vectors served as a negative control to exclude non-specific background fluorescence. Fluorescent signals were observed under a fluorescence microscope. Scale bar = 100 μm

To investigate the mechanistic role of the candidate gene, *HvSRP_Z7* was amplified and cloned into pGBKT7 to serve as the bait protein (pGBKT7-HvSRP_Z7), while *HvHor* was amplified and cloned into pGADT7 as the prey protein (pGADT7-Hor). All four strains were capable of growing on SD-TL medium(Figure. 6 A). In contrast, the strain transformed with pGBKT7-HvSRP_Z7 + pGADT7-Hor grew normally on all four media types: SD-TL, SD-TLH, SD-TLHA, SD-TLH+X-α-gal, and SD-TLHA + X-α-gal, and turned blue specifically on the media supplemented with X-α-gal. The negative control strain (pGBKT7-LaminC + pGADT7-LargeT) failed to grow on SD-TLH, SD-TLH supplemented with X-α-gal, SD-TLHA, and SD-TLHA supplemented with X-α-gal media. This result indicates that the selective pressure of the media was effective and that no cross-contamination occurred during the experimental procedure. Building upon this, the strain transformed with pGBKT7-HvSRP_Z7 + pGADT7 (empty vector) failed to grow on SD-TLH, SD-TLH supplemented with X-α-gal, SD-TLHA, and SD-TLHA supplemented with X-α-gal media. In contrast, the strain transformed with pGBKT7-HvSRP_Z7 + pGADT7-HvHor grew normally and produced a color change (turned blue) on all four media types: SD-TLH, SD-TLH + X-α-gal, SD-TLHA, and SD-TLHA + X-α-gal. These results demonstrate a specific interaction between the two proteins. The BiFC assay was performed by constructing and co-infiltrating *Nicotiana benthamiana* leaves. With HvSRP_Z7-YN and HvHor-YC vectors for co-expression. A specific YFP signal was detected in cells co-expressing HvSRP_Z7-YN and HvHor-YC, whereas no signal was observed in the negative control groups (Fig. 6B). This result provides evidence for the interaction between *HvSRP_Z7* and *HvHor*.

The BiFC assay was performed in *Nicotiana benthamiana* leaf epidermal cells, a heterologous system that may not fully recapitulate the cellular environment of barley endosperm. Hordeins are storage proteins that normally accumulate in endoplasmic reticulum (ER)-derived protein bodies in developing barley grains(Tanner et al. 2024). Consequently, their folding, trafficking, and stability may differ in the leaf system, which explains the relatively weaker fluorescence intensity observed in the experimental combination compared to the positive control. Therefore, it is important to emphasize that the interaction was considered valid based on the presence of a specific signal—which was consistently absent in all negative control combinations—rather than on absolute signal intensity(Kudla and Bock 2016; Liu et al. 2014).

## Discussion

Given that grain size is a key determinant of crop yield, elucidating its molecular regulatory mechanisms is crucial for advancing molecular design breeding in barley. This study utilized transcriptome data from barley varieties with contrasting grain sizes, combined with WGCNA, to identify key co-expression modules and candidate genes associated with grain development. Phenotypic dynamic analysis revealed that grain length and thickness showed significant differences between the tested cultivars at all filling stages, whereas differences in grain width only became apparent during the mid to late filling stages. This observed pattern may be attributed to the relatively lower number of DEGs associated with grain width regulation during the early filling phase. Our findings demonstrated that both radial and transverse expansion parameters collectively regulate grain morphogenesis, with radial expansion playing the dominant role. These findings provide new insights into elucidating the genetic basis of grain size in hull-less barley.

High-quality transcriptome data (Q30 > 95%) provided a reliable foundation for subsequent differential expression analysis and WGCNA. Differential expression analysis revealed dynamic transcriptional changes during barley grain development. The number of DEGs peaked at the mid-filling stage, with a subsequent decline during the late stage, suggesting this period as a critical regulatory window for grain size determination. Grain development has been classified into three distinct phases: the initial formation stage, the rapid dry matter accumulation stage, and the maturation stage(Du et al. 2019). The mid-filling stage, characterized by a linear increase in grain weight, represents a phase of active dry matter synthesis and accumulation. This heightened metabolic activity likely drives the extensive differential gene expression observed, thereby accounting for the notable peak in DEG numbers during this critical developmental window. WGCNA further identified a brown module that exhibited strong positive correlations with all three grain dimensions, showing correlation coefficients of 0.87 with grain length, 0.845 with grain width, and 0.913 with grain thickness. The brown module, containing 233 genes, was subjected to intramodular connectivity analysis to identify the top 30 hub genes. By intersecting these hub genes with the DEGs and performing functional annotation, we ultimately identified five candidate genes potentially involved in the regulation of grain development.

*HvSRP_Z7* is annotated as encoding a member of the serpin (serine protease inhibitor) family. Previous studies have revealed differential accumulation levels of this protein family among wheat germplasms with contrasting grain sizes(Zhang et al. 2015), with significantly higher abundance observed in small-grained varieties compared to large-grained ones, suggesting its potential involvement in the regulatory network governing grain development. Serine protease inhibitors exhibit functional diversity in plants, functioning not only as exogenous protease inhibitors involved in defense responses but also influencing protein stability through non-inhibitory functions, such as acting as molecular chaperones(Cohen et al. 2019). Both of these functions may be relevant to the regulation of grain size: if functioning as a protease inhibitor, it may interfere with storage protein processing or the programmed cell death process by inhibiting key proteases involved in endosperm development(Asqui et al. 2018; Lampl et al. 2013). If functioning as a molecular chaperone, it may induce endoplasmic reticulum (ER) stress by affecting the proper folding of storage proteins (e.g., B-hordein), thereby limiting endosperm cell expansion(Cohen and Fluhr 2018). Furthermore, serpin has been reported to be associated with β-glucan content(Li et al. 2018). However, current research on the influence of glucans on grain development has primarily focused on α-glucans(Trafford et al. 2013). No studies have directly demonstrated the involvement of β-glucan in regulating grain size. However, existing evidence indicates that β-glucan is a major component of the cell wall, and its metabolic dynamics may participate in the regulation of grain filling by affecting cell wall relaxation and expansion capacity(Nadiminti et al. 2024; Trafford et al. 2013). However, whether serpin directly participates in the regulation of grain size in crops remains unclear, and functional studies in this area are still limited. In the present study, the expression level of *HvSRP_Z7* was continuously up-regulated throughout the grain filling period, and was significantly higher in the small-grained cultivar than in the large-grained cultivar at the middle and late filling stages. Based on this, it is hypothesized that its expression level is negatively associated with grain size. *HvSRP_Z7* may restrict endosperm cell filling and expansion through one or more of the mechanisms discussed above. These include the inhibition of key proteases, interference with the proper folding of storage proteins, and potential effects on cell wall composition. However, an alternative interpretation of this observation exists. The elevated expression of *HvSRP_Z7* in the small-grained cultivar may reflect a compensatory response to limited grain filling, rather than directly restricting grain growth. Such compensatory mechanisms have been documented in other cereal species(Dong et al. 2019; Sanchez-Bragado et al. 2020). Among the five candidate genes selected for analysis, *HvEXPA* exhibited the highest expression level in qRT-PCR assays. However, its homologs in other crops, such as *Arabidopsis* and rice, have previously been demonstrated to be associated with grain size regulation. Therefore, *HvSRP_Z7* was prioritized as the core candidate gene for further functional investigation, and yeast two-hybrid (Y2H) and bimolecular fluorescence complementation (BiFC) assays were subsequently performed. The experimental results confirmed a physical interaction between *HvSRP_Z7* and the B-hordein-encoding protein (*HvHor*). B-hordein serves as the major storage protein in barley. Its homolog in wheat has been demonstrated to participate in the regulation of grain size formation(Yan et al. 2016). These interaction results further support the hypothesis that *HvSRP_Z7* may participate in the regulation of grain size by influencing storage protein deposition. Taken together, this study proposes that *HvSRP_Z7* may function as a negative regulator of grain development, with its underlying mechanism potentially involving the modulation of protease activity, facilitation of storage protein folding, or regulation of cell wall metabolism. Future studies employing genetic approaches (e.g., gene knockout or overexpression), in conjunction with cytological and biochemical analyses, will be essential to systematically elucidate its biological function.

The candidate gene *HvEXPA* is annotated as encoding an expansin protein. Expansins play a critical role in plant cell wall modification, and previous studies have demonstrated their important functions in grain development. For instance, overexpression of the sweet potato expansin gene *IbEXP1* in *Arabidopsis* has been shown to significantly increase grain size(Bae et al. 2014). Similarly, overexpression of *OsEXPA7* in rice has been shown to promote grain length and increase thousand-grain weight(Zhang et al. 2024). In these studies, expansins generally regulate grain size by mediating cell wall loosening and extensibility. However, in the present study, *HvEXPA* exhibited a sustained up-regulation throughout the grain filling process in barley, with expression levels peaking at the late filling stage. Furthermore, this gene exhibited significantly higher expression levels in the small-grained cultivar at both the middle and late filling stages. This expression pattern suggests a negative correlation between the expression level of *HvEXPA* and grain size, implying that its high expression may be associated with the formation of the small-grain phenotype. This seemingly paradoxical observation may be explained by several factors. Studies have indicated that the regulation of cell expansion by expansins exhibits spatiotemporal specificity. While moderate expression of expansins promotes cell elongation, excessive expression may disrupt normal growth through multiple mechanisms, including perturbation of cell wall remodeling, alterations in transcriptional regulatory networks, interference with hormone signaling (particularly gibberellin signaling), and disruption of cell wall differentiation and secondary wall formation, ultimately leading to impaired cell elongation(Ilias et al. 2019; Luan et al. 2022). Therefore, the relatively high expression level of *HvEXPA* in the small-grained cultivar may not be primarily involved in mediating cell expansion. Instead, it may indirectly or directly participate in regulatory pathways that restrict cell expansion. Based on this differential expression pattern, we hypothesize that *HvEXPA* may be involved in the negative regulation of grain size formation during the grain filling stage in barley. This hypothesis awaits further validation through functional experiments.

*HvSufS* is annotated as encoding a cysteine desulfurase. Studies have shown that in soybean, this enzyme does not directly regulate grain size. Instead, it participates in iron‑sulfur (Fe–S) cluster biosynthesis by catalyzing the release of sulfur from cysteine. As Fe–S clusters are essential cofactors for nitrogenase and components of the electron transport chain, their biosynthesis contributes to enhanced nitrogen fixation efficiency, thereby promoting plant growth and indirectly increasing seed size(Igiehon et al. 2021). This mechanism provides a new perspective for understanding the regulatory network of grain size in barley, suggesting that *HvSufS* may indirectly participate in grain development by influencing sulfur metabolism and energy utilization efficiency. In the present study, *HvSufS* exhibited distinct expression dynamics between large- and small-grained cultivars. In the small-grained cultivar, its expression peaked at the middle filling stage and subsequently declined. In contrast, expression in the large-grained cultivar continuously increased from the early filling stage and reached its maximum at the late stage. Notably, *HvSufS* expression levels were significantly higher in the large-grained cultivar at both the middle and late filling stages—approximately 6.7-fold and 46.75-fold higher, respectively. This expression pattern suggests a positive correlation between *HvSufS* expression and grain size, implying that its higher expression may be associated with the formation of the large-grain phenotype. This process could influence the efficiency of the mitochondrial electron transport chain and ATP generation capacity, thereby providing enhanced energy support for the grain filling process(Nath et al. 2016; Satyanarayan et al. 2021). In soybean, genes involved in iron‑sulfur cluster biosynthesis are activated within nodules. This activation is accompanied by enhanced Fe‑S enzyme activities and iron enrichment, providing essential energy and cofactor support for the nitrogen fixation process, thereby influencing nodule size(Qin et al. 2015). In summary, based on its molecular function, it is hypothesized that the cysteine desulfurase encoded by *HvSufS* may participate in iron‑sulfur (Fe–S) cluster biosynthesis, thereby influencing mitochondrial electron transport chain efficiency and ATP generation capacity. This process could consequently provide enhanced energy support for grain filling, thus contributing to the positive regulation of grain size. However, this hypothesis remains speculative at present. The precise function of HvSufS in barley, as well as its underlying regulatory mechanisms, awaits further validation through genetic and molecular approaches (e.g., gene knockout or overexpression).

The candidate gene *HvTSJT1* is annotated as encoding a stem-specific protein TSJT1. To date, the biological function and molecular mechanisms of this gene in crops have not been systematically characterized. Limited studies suggest that in sesame, this gene may influence grain size through the regulation of cell growth(Okuno et al. 2024). However, apart from that report, no other studies have confirmed a direct association between this gene and grain size regulation. Therefore, whether *HvTSJT1* participates in the regulation of grain development in barley remains unknown. In this study, the expression of *HvTSJT1* peaked at the late filling stage in both large- and small-grained cultivars. Highly significant genotypic differences were observed in its expression levels at both the middle and late filling stages. Notably, the relative expression level of this gene in the small-grained cultivar was 9.7 times higher than that in the large-grained cultivar at the middle filling stage. This difference dramatically widened to 50.36 times at the late filling stage. This extremely pronounced and continuously widening expression pattern strongly suggests a negative correlation between the expression level of *HvTSJT1* and grain size, implying that its remarkably high expression may be associated with the formation of the small-grain phenotype. Based on the aforementioned expression characteristics and the limited evidence from sesame, we hypothesize that *HvTSJT1* may participate in the grain size regulatory network in a negative regulatory manner during the middle and late grain filling stages in barley, thereby contributing to the observed variation in grain size between cultivars. However, given the extremely limited functional studies currently available on this gene, its specific mechanism of action remains unclear. This hypothesis awaits further validation through subsequent functional experiments (e.g., gene knockout or overexpression).

*HvSCPL* is annotated as encoding serine carboxypeptidase II. This protein family plays an important role in grain development in gramineous crops. Studies have shown that serine carboxypeptidase in wheat can increase grain size by promoting cell division and elongation(Mangini et al. 2021). In rice, *GS5*, which encodes a serine carboxypeptidase, has been identified as a key regulator of grain size. Polymorphisms in its upstream regulatory region influence grain size by affecting the gene’s response to abscisic acid (ABA) and its expression levels in young panicles (with *GS5-1* exhibiting higher expression than *GS5-2*)(Li et al. 2011; Xu et al. 2015). These studies suggest that members of this protein family generally function as positive regulators involved in grain development. However, the expression pattern of *HvSCPL* observed in this study contrasts with these findings. Its expression peaked at the middle filling stage and subsequently declined, with expression levels in the small-grained cultivar being significantly higher than those in the large-grained cultivar at both the middle and late filling stages (9.7-fold and 50.36-fold, respectively). This expression pattern suggests that *HvSCPL* expression may be negatively associated with grain size. Given that *HvSCPL* is an ortholog of rice *GS5*, the contrasting expression-phenotype association between the two genes may stem from the following factors. First, species-specific differences in regulatory networks may exist. Barley and rice exhibit substantial differences in endosperm development and storage reserve accumulation. Specifically, grain development in barley is highly dependent on the accumulation of hordeins and the formation of protein bodies. As a proteolytic enzyme, serine carboxypeptidase may therefore play a role in regulating grain filling by influencing the processing and deposition of these storage proteins(Diaz-Mendoza et al. 2019; Gasparis and Miloszewski 2023). Therefore, the function of *HvSCPL* in barley may differ from that of its ortholog *GS5* in rice. Second, differences in developmental stage sampling may contribute to this discrepancy. The sampling time points in the present study (2, 3, and 5 weeks after anthesis) covered the early, middle, and late grain filling stages. However, a finer temporal dynamic analysis (e.g., sampling at 5-day intervals after anthesis) might reveal distinct expression patterns of *HvSCPL* at earlier developmental stages. Third, functional divergence at the isoform level may exist. The expression analysis in this study was conducted at the level of total transcript abundance, which does not distinguish between potentially different splice variants. The serine carboxypeptidase family comprises numerous members, and different isoforms may exhibit functional divergence. It is possible that the expression patterns of certain isoforms differ from that of the total transcript level, or that specific isoforms display cultivar-specific distribution(Ren et al. 2021). Moreover, interference from genetic background effects may also play a role. The two cultivars used in this study (Heiseyeqingke and Jianglinhuang) possess distinct genetic backgrounds and are not near-isogenic lines (NILs) or recombinant inbred lines (RILs). Grain size, as a complex quantitative trait, is co-regulated by multiple genetic loci. Genes such as *GS3*, *GW2*, and *GS5* in rice exhibit complex genetic interactions and background-dependent effects(Lu et al. 2013; Wang et al. 2024). Therefore, the potential influence of other factors within the genetic background on the observed expression-phenotype association cannot be completely excluded. Based on the aforementioned expression characteristics and functional clues, we hypothesize that *HvSCPL* may participate in the grain size regulatory network in barley in a negative regulatory manner. Its mechanism of action may differ from that of the known positive regulator *GS5*, potentially involving processes such as storage protein metabolism, cell wall modification, or hormone signaling pathways. However, this hypothesis remains speculative at present. The precise function of *HvSCPL* in barley and its potential regulatory mechanisms await further validation through subsequent functional experiments.

It is worth noting that the two cultivars used in this study, Heiseyeqingke and Jianglinhuang barley, possess distinct genetic backgrounds. And they are not near-isogenic lines (NILs) or recombinant inbred lines (RILs). Therefore, although differential expression of the candidate genes (e.g., *HvSRP_Z7*) was observed, the potential influence of other genetic factors on grain phenotypes cannot be entirely excluded. Future studies employing near-isogenic lines or transgenic experiments will be necessary to further validate the direct roles of these genes in grain size determination.

## Conclusion

This study systematically investigated the regulatory mechanisms of grain development through integrated transcriptome and WGCNA using two barley cultivars with significant differences in grain traits. RNA-seq analysis identified 6,438 DEGs, with their abundance peaking during the mid-filling stage, indicating this phase represents a critical period for grain formation. WGCNA further identified a core module significantly associated with grain size traits. Subsequent analysis, based on the topological overlap matrix and intramodular connectivity, combined with intersection analysis using the DEGs, ultimately pinpointed five key candidate genes. RT-qPCR validation confirmed that all these genes exhibited stage- and cultivar-specific expression patterns. Specifically, *HvSufS* likely functions as a positive regulator promoting grain development, whereas *HvSCPL*, *HvTSJT1*, *HvEXPA*, and *HvSRP_Z7* may act as negative regulators constraining grain size. Y2H and BiFC experiments demonstrated that *HvSRP_Z7* interacts with grain size-controlling proteins. These findings provide new evidence for elucidating the molecular network of barley grain formation and establish a theoretical foundation for developing large-grain germplasm resources through molecular breeding.

## Supplementary Information

Below is the link to the electronic supplementary material.

Supplementary Material 1

Supplementary Material 2

Supplementary Material 3

Supplementary Material 4

Supplementary Material 5

Supplementary Material 6

Supplementary Material 7

Supplementary Material 8

Supplementary Material 9

## Data Availability

Raw data have been deposited to National Center for Biotechnology Information (NCBI) under the BioProject number PRJNA1232405 (https://dataview.ncbi.nlm.nih.gov/object/PRJNA1232405?reviewer=5epc2u659omr96evi6h4d5bnds).
